# Paris Hospitals

**Published:** 1919-01

**Authors:** T. D. Boulanger


### PARIS HOSPITALS

Prepared by Captain T. D. Boulanger, Liaison Officer in Department of Medical Research and Intelligence, American Red Cross in France.

### Introduction.

Contrary to the custom of hospitals of other countries, which are most often kept up by their own endowment, private benefaction or gifts, and without municipal support, and each managed according to its own rules, the Hospitals of the City of Paris are dependent on the city government, namely the Administration Générale de l'Assistance Publique. They are founded on the principle of free and unrestricted admission of the poor.

In addition to Hospitals and Almshouses, the City of Paris supports a large number of other benefactions : Bureaux de Bienfaisance, Dispensaires, Consultations des Nourrissons (bringing-up 55,000 foundlings). The Administration Générale de l'Assistance Publique was established in 1802.

The teaching of medicine in France is in the hands of the State, which has seven Faculties in different Centers : Paris, Lyon, Bordeaux, Nancy, Lille, Toulouse, Montpellier. In Paris, besides the Faculté, there is the Val-de-Grace, a military medical school; the naval branch is in Bordeaux. The Université de Paris has no Hospital of its own, the clinics being furnished by all the Hospitals and Institutions under the Administration de l'Assistance Publique. This feature is of the greatest benefit both to the patients and to medical teaching and science. All hospital physicians, surgeons and specialists are chosen after competitive examination. The Assistance Publique is closely connected with the Faculté de Médecine and provides that every hospital is a suitable institution for teaching; numerous clinic rooms and halls are specially equipped for lectures, "Cliniques de la Faculté". The École de Médecine is situated on Boulevard St. Germain and Rue de l'École de Médecine (main entrance) and has the largest teaching personnel of any medical school in France — a total of 81. A school for nurses, École des Infirmières de l'Assistance Publique, was founded in 1907, and is located at the Hospice de la Salpêtrière.

The Conseil Municipal de Paris gives a certain sum yearly to the Assistance Publique.

We are indebted to the Directeur de l'Administration Générale de l'Assistance Publique de Paris, Monsieur G. Mesureur, for the valuable assistance given to us in the compilation of this booklet.

Note. — A card of admission to the Hospitals of Paris may be obtained from the Administration Générale de l'Assistance Publique, N° 3, Avenue Victoria, Paris (1er). Métro Station " Cité ", or at the office of the Department of Research and Intelligence, Room 429, American Red Cross Headquarters, 2, Place de Rivoli, Paris.

### Hotel-Dieu.

Parvis Notre-Dame. Subway, Metro station " Cité ".

The old Hotel-Dieu, the origin of which is so very ancient that it is not well-known, was pulled down in 1877 and new buildings were constructed from 1865 to 1877 on another part of the Ile de la Cité, close to the new Place de l'Hôtel-de-Ville. The old Hotel-Dieu was built on both banks of the small river and on two bridges. The New Hotel-Dieu shows the way in which a hospital was built thirty years ago. Every visitor should see the oldest church in Paris, the church of the Hotel-Dieu which belongs to the Assistance Publique: Saint-Julien-le-Pauvre, reached by the Rue de la Bucherie and the small lane Saint-Julien-le-Pauvre.

Hotel-Dieu admits patients with all general diseases, with departments of Ophthalmology, Neurology and Obstetrics. It has 885 beds, and 15,000 patients are received yearly.

Staff : *Physicians* : MM. Chantemesse, A. Petit, Gilbert, Prof. de Clinique, Roger, Dalche, Caussade.
*Surgeons* : MM. Hartmann, Prof. de Clinique; Potherat.
*Ophthalmologist* : M. de Lapersonne, Prof. de Clinique.

### La Pitié.

83, Boulevard de l'Hôpital. Subway, Metro station " St. Marcel ".

Notre Dame de Pitié was constructed during the 17th century by Louis XIII for the aged poor and homeless, and reconstructed by Louis XIV. It became an annex of the Hotel-Dieu from 1809, and since 1816 a general hospital with medical and surgical departments. La Pitié was reconstructed ten years ago, and is to-day one of the most modern hospitals in Paris. A nurses' training school is also connected with La Pitié, 994 beds.

Staff : *Physicians* : MM. Babinski, Lion, Claisse, Thirolois, Enriquez, Josué.
*Surgeons* : MM. Walther, Arrou, Thiéry.
*Obstetrician* : M. Potocki.

### Necker.

151, Rue de Sèvres. Subway, Nord-Sud station "Falguière".

The present site of the Hospital was occupied by a Benedictine Convent, leased by Louis XIV and named after Mme. Necker, the first director. 475 beds. A painting by Bisson representing an operation by Guyon is seen in the museum of the genito-urinary clinic.

Staff : *Physicians* : MM. Barth, Achard, Renon.
*Surgeons* : MM. Delbet, Prof. de Clinique; Routier, Legueu.

### Saint-Antoine.

184, Rue du Faubourg Saint-Antoine. Subway, Metro station "Reuilly".

The hospital of Saint-Antoine was established in the buildings of the ancient abbey Saint-Antoine-des-Champs, founded about 1198. The buildings were enlarged by wings. Mr. Moiana, by his bequest, enabled the Assistance Publique to build a new pavilion close to the Boulevard Diderot (foundation Moiana); lately a new lying-in ward has been erected with all modern improvements. The oldest wards were pulled down and the out-patient department rebuilt. Special attention is given to the X-Ray department for the examination of patients, and mainly to the X-Ray and radium treatment of cancer, with histological rooms for research workers, museum, etc. The beds are 900 in number, with numerous extra beds in winter. At Boulevard Diderot the medical section, under Dr. Beclère, includes a Laboratory of Medical Radiology, where X-Rays and radium are used. A very complete library on radiology and radium-therapy, and a museum containing a great number of casts and photographs have been collected to help in the study of those branches.

Staff : *Physicians* : MM. Chauffard, Prof. de Clinique; Sireday, Beclère, Vaquez, Le Noir, Mosny, Clause.
*Surgeons* : MM. Lejars, Ricard.
*Oto-Rhino-Laryngologist* : M. Lermoyez, Méd. des Hôpitaux.

### Cochin.

47, Rue du Faubourg-Saint-Jacques. Subway, Metro station "St.-Jacques".

The Hospital Cochin was established in 1782 by the generous endowment of Cochin, vicar of Saint-Jacques-du-Haut-Pas; but it consisted only of the small block on the Rue Saint-Jacques. The grounds were used for the required enlargements, and wooden wards were built on account of an epidemic twenty years ago.

Nevertheless to-day there stands a well-equipped modern building, and the whole hospital, with the late Ricord Hospital (venereal diseases, men) is rebuilt with every requirement for the treatment of every kind of disease, for in and out-patients.

The hospital with the annex (late Ricord) contains 817 beds which are allotted as follows : — medicine 229, surgery 271, venereal diseases 317. A museum for venereal diseases contains many valuable specimens.

Staff : *Phycicians* : MM. Oettinger, Widal, Gandy, Quayrat, Fournier.
*Surgeons* : MM. Schwartz, Quenu, Prof. de Clinique; Faure, Michon.

### Beaujon.

208, Rue du Faubourg-Saint-Honoré. Subway, Metro station " Alma ".

Built by Beaujon in 1784. Contains 610 beds.

Staff : *Physicians* : MM. Debove, Prof. de Clinique; Alb. Robin, Prof. de Clinique Thérapeutique; Faisans.
*Surgeons* : MM. Bazy, Tuffier, Michaux.
*Ophthalmologist* : M. Terrien.
*Obstetrician* : M. Ribemont-Dessaignes, Prof. de Clinique.

### Lariboisière.

2, Rue Ambroise Paré. Subway, Metro station " Barbès ".

The hospital Lariboisière was established partly by the generous endowment of the Comtesse de Lariboisiere, in 1854, for a service of 600 beds. The wards were arranged in pavilions on each side of large grounds. The Hospital Lariboisière, close to the Gare du Nord, standing in a most active and populous part of Paris, receives yearly more than 20,000 bed patients and 12,000 out-patients. It admits every kind of general disease mentioned below, including confinement cases; a school for the training of nurses (for out-scholars only) is annexed.

Lately a new block, equipped with all modern requirements, was built for genito-urinary cases and named Service Civiale.

Staff : *Physicians* : MM. Brault, Gaillard, Le Gendre, Florand.
*Surgeons* : MM. Marion, Chaput.
*Ophthalmologist* : M. Morax.
*Oto-Rhino-Laryngologist* : M. P. Sebileau.

### Tenon.

4, Rue de la Chine. Subway, Metro station " Place Gambetta ".

The old Menilmontant hospital (1878) became the Tenon in 1879. 975 beds.

Staff : *Physicians* : MM. Menetrier, Klippel, Parmentier, Gouget, Macaigne, Carnot, Laffitte, Lesne.
*Surgeons* : MM. Riche, Robineau, Lenormant.

### LAËNNEC.

42, Rue de Sèvres. Subway, Metro station "Sèvres".

Built in 1635, it was at first known as Hospital for The Incurable. It became a general hospital in 1878 and was named Laënnec. Tuberculous soldiers are treated there. Diagnosis by X-rays and all modern equipment.

Staff : *Physicians* : MM. Küss, Bernard, Rist, Dupré.
*Surgeons* : M. Desmarest.
*Radiologist* : M. Maingot.
*Ophthalmologist* : M. Rochon-Duvigneaud.
*Oto-Rhino-Laryngologist* : M. Lombard.

### BICHAT.

Boulevard Ney. Subway, Nord-Sud station " Porte St.-Ouen ".

Opened in 1882 by the transformation of old barracks No. 39, to which many additions have been made.

Staff : *Physicians* : MM. Talamon, Bruhl.
*Surgeon* : M. Launay.

### BROUSSAIS.

96, Rue Didot. Subway, Metro station " Porte d'Orléans ".

Built in 1883 for the treatment of cholera, and continued as a general hospital after the end of the epidemic.

Staff : *Physicians* : MM. Berge, Dufour.
*Surgeon* : M. Auvray.

### BOUCICAUT.

62, Rue de la Convention. Subway, Metro station " Beaugrenelle ".

Mme. Boucicaut having made a large general bequest to the Assistance Publique, a modern hospital was built in 1897 in Grenelle's populous ward, and visitors may see at Boucicaut Hospital the latest and most comfortable buildings as well as modern fittings. The system of small pavilions, isolated on a large ground, having only a small number of beds, and completely separated, has been applied as much as possible. The patients are provided with all conveniences: dining-hall, winter-garden, etc. An underground way connects every pavilion with admission office, kitchen, etc. The hospital contains 254 beds.

Staff : *Physicians* : M. Letulle.
*Surgeon* : M. Demoulin.
*Obstetrician* : M. Lepage.

### Andral.

Boulevard McDonald. Subway, Metro station " Porte de la Villette ".

Old convent, successively used as hospital, surgical store, special services, etc., and was definitely turned into a general hospital in 1885, and named Andral.

Staff : *Physicians* : MM. De Massary, Apert.

### Saint-Louis.

40, Rue Bichat. Subway, Metro station " Republique ".

Saint-Louis is an old and architecturally interesting building and owes its origin to the plague. King Henry IV established it towards 1610 under the supervision of the famous architect Claude Vellefaux, and Saint-Louis stays as a splendid model of ancient architecture. It provides treatment for every kind of cutaneous and venereal disease, and is famous for the Museum and the Bibliotheque Feulard. From all points of France and colonies abnormal cases are sent to Saint-Louis and the visitors may see the lepers, the light treatment of lupus, X-ray department and electric treatment (high frequency) of cancer, etc.

Staff : *Physicians* : MM. Gaucher, Prof. de Clinique; Darier, Balzer, Brocq, Thibierge, Sabouraud, Jeanselme.
*Surgeons* : MM. Rochard, Rieffel, Morestin.
*Obstetrician* : M. Damelin.
Childrens' Service : *Surgeon for children* : M. Baudet.
*Pediatrician* : M. Renault (Jules).

### Broca.

111, Rue Broca. Subway, Metro station " Glacière ".

Hopital Broca, called De Lourcine up to 1893, was opened in 1836 on the grounds of the old Cordelières Convent, founded about 1284 by Marguerite de Provence, Saint Louis' sister. It was first devoted to the treatment of venereal diseases in women exclusively. Since 1898 skin and gynecological departments have been added and they have one of the most modern equipments in the city. There is a very interesting museum.

Staff : *Physicians* : MM. Jeanselme, Hudelo.

### Claude Bernard.

Porte d'Aubervilliers. Subway, Nord-Sud station " Porte de la Chapelle ".

Isolation hospital for infectious diseases. 307 beds.

Staff : *Physician* : M. Teissier.

### LYING-IN HOSPITALS

### Maternité.

119, Boulevard Port-Royal. Subway, Metro station " St.-Jacques ".

The Maternité was established about 1814 in the buildings of the late convent of Port-Royal, and the old cloister of the Jansenists is yet to be seen. But the old buildings are to-day completed by a new block, equipped with all modern requirements and the service consists of 360 beds. The old buildings were modernized in their fittings to receive a home for the training of 100 midwives, and no school in France surpasses this Maternity School. With two other hospitals situated close by, the Clinique Baudelocque and the Clinique Tarnier, this lying-in service handles more than 6,000 accouchements yearly; 20 town midwives receive pregnant women in 120 beds when they cannot be admitted to the hospital.

Staff : *Physician* : M. Garnier.
*Chief Obstetrician* : M. Bonnaire.
*Asst.* » : M. Brindeau.

### Baudelocque.

125, Boulevard Port-Royal. Subway, Metro station " St.-Jacques ".

Adjoining the above, the Clinique Baudelocque was opened in 1886 and has since a gynecological department. 188 beds.

Staff : M. Couvelaire, *Prof. de Clinique Obstétricale.*

### Clinique Tarnier.

89, Rue d'Assas. Subway, Nord-Sud station " Notre-Dame-des-Champs ".

Opened in 1881, this clinique was named after the great obstetrician soon after his death in 1887. 210 beds.

Staff : M. Bar, *Prof. de Clinique Obstétricale.*

Both of the above institutions are under the Direction of the Maternité.

La Pitié, Charité, St. Antoine, Beaujon, Lariboisière, Tenon,

Boucicaut and St. Louis, have lying-in departments. Three out-of-town places at Roubaix, Mont St. Augnau and Chalon-sur-Saône also have lying-in departments.

### HOSPITALS FOR CHILDREN

### Enfants Malades.

149, Rue de Sèvres. Subway, Nord-Sud station " Falguière ".

Bought by the curate of St. Sulpice, 1732, this hospital at first received women and young girls of the parish and is still known under the name of " Enfant Jesus ". This hospital, together with those Trousseau, Bretonneau and Herold is exclusively reserved for sick children of both sexes, from two to fifteen years of age.

Staff : *Physicians* : MM. Hutinel, Prof. de Clinique; Comby, Richardière, Marfan, Méry, Aviragnet.
*Surgeons* : MM. Kirmisson, Prof. de Clinique; Broca.
*Ophthalmologist* : M. Poulard.

### Trousseau.

158, Rue Michel-Bizot. Subway, Metro station " Porte de Vincennes ".

365 beds.

Staff : *Physicians* : MM. Netter, Triboulet.
*Surgeon* : M. Savariaud.

### Bretonneau.

2, Rue Carpeaux. Subway, Nord-Sud station " La Fourche ".

261 beds.

Staff : *Physicians* : MM. Guinon, Boulloche.
*Surgeon* : M. Ombredanne.

### Herold.

5, Place du Danube. Subway, Metro station " Place du Danube ".

258 beds.

Staff : *Physicians* : MM. Barbier, Lesage.

### Berck-sur-Mer (Pas-de-Calais).

After eight years of successful results in the treatment of scrofulous children at the seashore, the Assistance Publique founded a large institution at Berck-sur-Mer, known as the Hopital Maritime, for the exclusive treatment of those conditions. 1019 beds.

Staff : *Physician* : M. Ménard.

*Assistant* : MM. Calve et Andrieu.

### Asile pour Enfants (Hendaye).

Situated on the Bay of Biscay, for the treatment of anemic conditions in children. 644 beds.

Staff : *Physician* : M. Coronado.

### Hospice des Enfants Assistés.

74, Rue Denfert-Rochereau. Subway, Metro station " Denfert-Rochereau ".

For foundlings from birth to 12 years of age. 865 beds.

Staff : *Physician* : M. Variot.

*Surgeon* : M. Jalaguier.

### SPECIAL HOSPITALS

### Tuberculosis.

Dispensaire Léon-Bourgeois, 65, Rue Vaneau.
Laennec, 42, Rue de Sèvres; 225 beds. MM. Kuss, Maingot.
Brévannes (Seine-et-Oise) 148 beds.

### Ophthalmology.

Hôtel-Dieu, Lariboisière, Laennec.

### Oto-Rhino-Laryngology.

St.-Antoine, Lariboisière, Laennec.

### Genito-Urinary.

Necker, Lariboisière.

### NERVOUS DISEASES

### Salpêtrière.

It contains an almshouse for old and infirm females, an insane hospital, an infirmary for old people, wards for nervous diseases, a hospital for paralyzed and epileptic, an asylum for abnormal, idiotic and epileptic children, a reformatory for undisciplined girls (foundlings), besides medical and surgical wards and an outpatients department. The old buildings date from the XVIIth century, and the Salpêtrière was used as an almshouse for women, and as a prison for prostitutes.

The Salpêtrière was the center of Charcot's neurological activity. He gave his demonstrations and clinics there for many years. He was the founder of the University Neurological Department of which Professor Pierre Marie is now the head. Charcot's statue can be seen there. 3,858 beds for patients and a total of 6000 people within its walls.

There is also a school for nurses (day scholars) ; home for the training of nurses ; X-ray department ; electric treatment department.

Staff : *Physician* : M. Pierre Marie.
*Surgeon* : M. Gossel.
*Alienists* : MM. Chaslin, Seglas, Nageotte.

### Bicêtre.

78, Rue du Kremlin. Bicêtre (Seine).

Bicêtre, which dates back to 1236, became a pauper asylum in 1657, with a department for nervous diseases. Like the Salpêtrière, this institution is for aged paupers, blind, epileptics, incurables and nervous diseases. 3217 beds. Men only.

Staff : *Surgeon* : M. Dejarier.
*Alienists* : MM. Sougues, Roubinovitch, Riche.

### Val-de-Grace

277, Rue St-Jacques. Subway, Metro station " St-Jacques ".

The Val-de-Grace medical school and hospital are installed in an old Benedictine Convent built by Queen Anne of Austria to thank God for the birth of a son (Louis XIV, 1638).

The church built by Mansard and Leduc recalls, on a smaller scale, St.-Peter's of Rome. L'École d'Application du Service de Santé Militaire was organized in 1852 to prepare physicians and surgeons for military service. Due to war activities, the Val-de-Grace is to-day one of the most prominent centers of clinic and teaching in France.

The hospital has 1,100 beds reserved for army cases. One of the main features of the institution is the new Museum, under the direction of MM. Jacob and Pascal. It contains a great number of invaluable anatomo-pathological specimens of the present war. It is the historical Museum of the Service de Santé.

The Bacteriological Laboratory is directed by M. Besson.

A large Laboratory preparing anti-typhoid vaccines and making all the serotherapeutic researches for the army is directed by M. Vincent.

A very complete Library, containing French and foreign literature, is accessible to all physicians.

### Staff of the Hopital Militaire d'Instruction du Val-de-Grace.

*Surgery, General* : MM. Jalaguier, Sencert, Nageotte, Abadie, Bouffe-de-St-Blaize.
*Ophthalmologist* : M. Kalt.
*Oto-Rhino-Laryngologist* : M. Luc.
*Face Surgery and Face Restoration* : M. Morestin.
*Physicians* : MM. Chauffard, Richardière, Caussade, Florand.
*Neurology* : MM. Briand, Dupré, Roubinovitch, de Fleury, Delmas, Filassier.
*Tuberculosis* : M. Pissavy.
*Stomatology and Maxillo-facial prothesis* : M. Frem.
*Radiology* : M. Beclère.
*Electrotherapy* : M. Delille.
*Mecanotherapy* : M. Kouindjy.
*Ocular Prothesis* : M. Coulomb.

### Institut Pasteur.

25 Rue Dutot. Nord-Sud : Pasteur.

Pasteur's first laboratory was in the garret of Ecole Normale and occupied but a few square meters of space. After the report of his work on Rabies, the Academie des Sciences started a subscription which brought 2 1/2 million francs with which the Vaugirard place was erected. After Roux's communication at the Budapest Congress on the serum treatment of diphtheria, in 1894, another subscription started by " Le Figaro " brought one million francs, with which the great stables of Garches were built on the grounds loaned by the State. A great amount of private donations have enabled the Institute to become one of the largest and best equipped in the world. Branches have been established in almost every part of the world; the most important being at Lille (Dr. Calmette), Constantinople (Dr. Nicolle), Tunis (Dr. Lois), Saigon, Senegal, Madagascar, Annam (Dr. Yersin).

*Bacteriological Institute* : Three large buildings erected on Rue Dutot. Pasteur, who lived in the front part of the building, has been buried there and a magnificent tomb has been erected by Mrs. Pasteur and her children. A fine library on the first floor also contains a bust of Pasteur. On the ground floor are the Rabies department, where patients are inoculated every day, a large lecture hall, and large laboratories where vaccines and serums are prepared. There also all experiments on animals are made. The latter are kept in large stables back of the buildings. Since 1885, over 30,000 persons have been inoculated against rabies. A statue of a shepherd wrestling with a dog has been erected in the foreground to commemorate Pasteur's first inoculation against rabies. This shepherd, Jupille, is still concierge at the Institute. The department of technical microbiology is under Dr. Roux's direction. Two series of lectures (48 lessons each), are given every year — November and February — and are attended by a large number of scientific men. This department is also famous for Metchnikoff's great work on phagocytosis and technical microbiology, and Laveran's discovery of the malarial hematozoa.

*Institute of Serotherapy.* Preparation of various sera : antidiphtheric, antitetanic, antistreptococcic, antiplague, etc., under the direction of Dr. Roux. Large stables at Garches and Alleray St., where horses are immunized and serum collected. These sera are distributed to the State, Army and put on sale.

*Biological Chemistry.* Well equipped laboratories for biochemical research under Mr. Duclaux, professor of bio-chemistry at the Sorbonne, of which this is a department. Examination of all biological and pathological products.

*Department of Fermentations.* Teaching of scientific processes applied to industry : brewing, distilling, manufacture of yeast, wine, cider etc. A series of miniature factories is used for this purpose.

*Agricultural Chemistry.* Study of the physiology and pathology of the vegetable kingdom.

### Pasteur Hospital.

211 Rue de Vaugirard.

Founded in 1894 after the Budapest report on antidiphtheric serumtherapy. Two very modern and well equipped buildings for the treatment of diphtheria and other contagious diseases. Free consultation department and 100 beds, each being in a glass room with 2 doors : one on the porch and the other on the inside hall. Great care has been taken for perfect disinfection of the rooms. There is a number of smaller buildings used for isolation purposes. During the war MM. Martin, Veillon, Dorre, Mauny and Joubert have been in charge of the hospital.

